# Computational assessment of visual coding across mouse brain areas and behavioural states

**DOI:** 10.3389/fncom.2023.1269019

**Published:** 2023-10-13

**Authors:** Yizhou Xie, Sadra Sadeh

**Affiliations:** Department of Brain Sciences, Imperial College London, London, United Kingdom

**Keywords:** visual coding, decoding, behaviour, encoding, mouse visual cortex, neural activity

## Abstract

**Introduction:**

Our brain is bombarded by a diverse range of visual stimuli, which are converted into corresponding neuronal responses and processed throughout the visual system. The neural activity patterns that result from these external stimuli vary depending on the object or scene being observed, but they also change as a result of internal or behavioural states. This raises the question of to what extent it is possible to predict the presented visual stimuli from neural activity across behavioural states, and how this varies in different brain regions.

**Methods:**

To address this question, we assessed the computational capacity of decoders to extract visual information in awake behaving mice, by analysing publicly available standardised datasets from the Allen Brain Institute. We evaluated how natural movie frames can be distinguished based on the activity of units recorded in distinct brain regions and under different behavioural states. This analysis revealed the spectrum of visual information present in different brain regions in response to binary and multiclass classification tasks.

**Results:**

Visual cortical areas showed highest classification accuracies, followed by thalamic and midbrain regions, with hippocampal regions showing close to chance accuracy. In addition, we found that behavioural variability led to a decrease in decoding accuracy, whereby large behavioural changes between train and test sessions reduced the classification performance of the decoders. A generalised linear model analysis suggested that this deterioration in classification might be due to an independent modulation of neural activity by stimulus and behaviour. Finally, we reconstructed the natural movie frames from optimal linear classifiers, and observed a strong similarity between reconstructed and actual movie frames. However, the similarity was significantly higher when the decoders were trained and tested on sessions with similar behavioural states.

**Conclusion:**

Our analysis provides a systematic assessment of visual coding in the mouse brain, and sheds light on the spectrum of visual information present across brain areas and behavioural states.

## Introduction

The pattern of neural activity elicited by a visual stimulus depends on the object or scene being viewed. Past studies have explored how the brain translates such diverse visual stimuli into neural responses (DiCarlo et al., 2012; Ki et al., 2021). The question of visual coding and its relation to neural activity patterns can be studied from encoding point of view, namely how external stimuli determine neural responses. Different visual stimuli are processed within the visual system, leading to brain activity patterns related to the observed object or scene, but they are also influenced by internal states, such as arousal (Mickley Steinmetz and Kensinger, 2009; McGinley et al., 2015; Vinck et al., 2015), attention (Maunsell, 2015; Huang et al., 2023), or changes in behavioural states like running (Niell and Stryker, 2010; Vinck et al., 2015; Sadeh and Clopath, 2022). In particular, the modulation of neural activity by running may either enhance or suppress the responses of neurons to visual stimuli, with the exact effect depending on different parameters like the specific area involved (Nasr et al., 2015; Dadarlat and Stryker, 2017; Christensen and Pillow, 2022).

Due to the complex interaction of the external and internal parameters, it remains an open question how visual stimuli are encoded in different brain regions and under different behavioural states (Vinck et al., 2015; Stringer et al., 2019; Lazar et al., 2021). This question can be posed from a decoding point of view: given the pattern of neural activity recorded from the brain, how accurately the visual stimuli can be predicted or distinguished from each other? It is now possible to address this question more rigorously, thanks to recent technological developments that allow us to record and analyse neuronal activity from large-scale populations of neurons, while simultaneously quantifying different aspects of animals’ behaviour (Jun et al., 2017; Steinmetz et al., 2021).

Here, we used standardised publicly available datasets from the Allen Brain Institute, to evaluate the computational capacity of decoders to extract visual information across mouse brain regions and under different behavioural states. We focused on Neuropixels datasets, which allowed us to analyse large-scale population activity at precise temporal resolution and to link it with various behavioural measures, across a large number of animals. Specifically, we focused on analysing natural movie stimuli and the locomotion state of the animals to elucidate visual coding. We performed a systematic decoding survey, whereby the same decoding approaches were applied to neural activity patterns in response to multiple repeats of the same natural movie. By reconstructing decoder ‘receptive fields’, our approach allowed us to visualise and analyse the low-dimensional projections of the visual coding space from high-dimensional neural activity patterns. We further employed encoding models (generalised linear models) to predict the activity of individual units based on both the stimulus and behavioural parameters, and to quantify their interactions. Our study suggests a systematic approach to analysing visual coding across brain areas and behavioural states.

## Results

### Decoding natural images from neural activity

**Figure 1:**
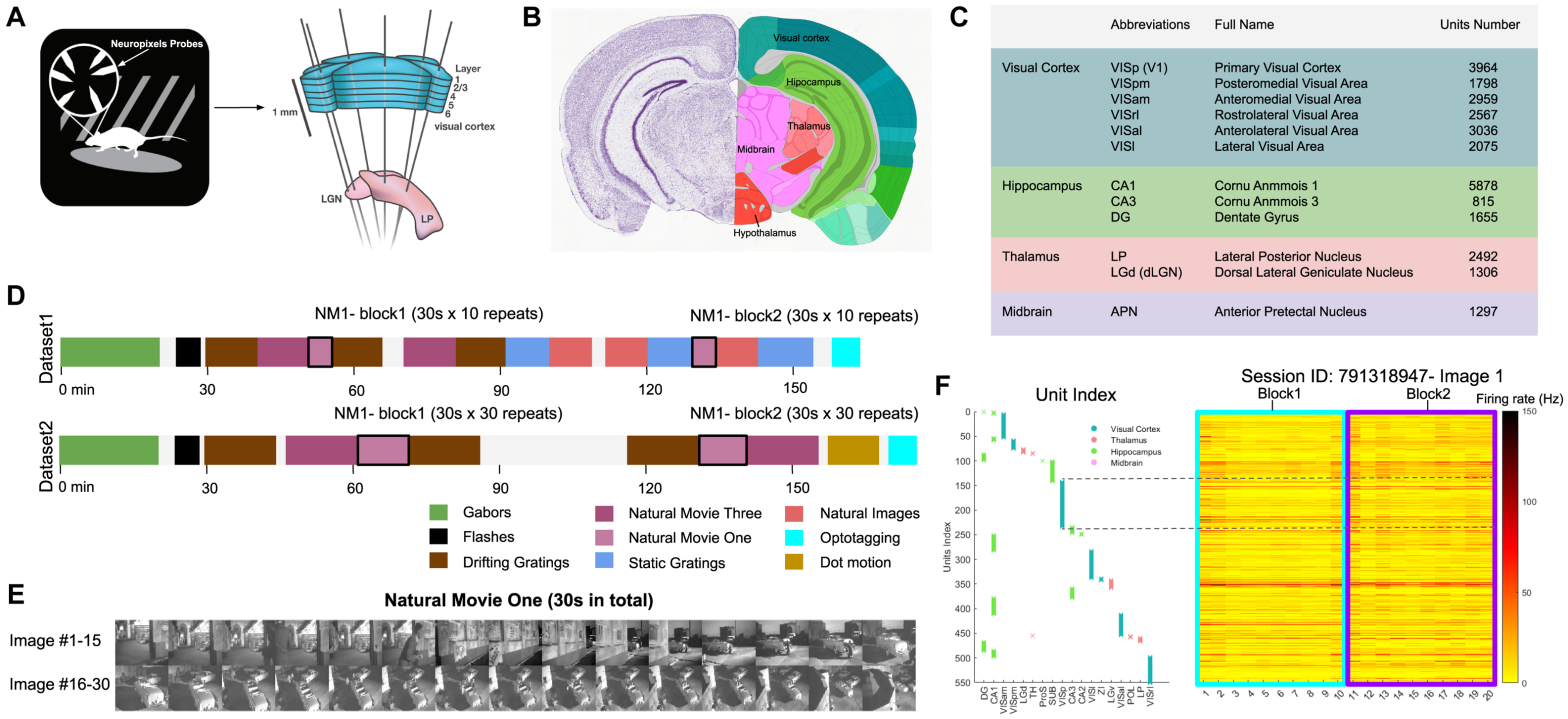
Overview of the dataset from Allen brain observatory, Neuropixels visual coding. **(A)** Data recording setup. Mice are head-fixed and can move on a rotating platform. Visual stimuli are displayed on a monitor, and neural activity is captured using six Neuropixels probes, targeting regions including the visual cortex, thalamus, midbrain, and hippocampus. **(B)** The adult mouse brain atlas. The main brain regions recorded during the recording session are shown, including the visual cortex, hippocampus, thalamus, and midbrain. Adapted from Allen Mouse Brain Atlas, https://atlas.brain-map.org/atlas. **(C)** Abbreviations and full names of some brain regions in the dataset. To facilitate better understanding, we also provide some alternative abbreviations that are more commonly used (such as V1 for primary visual cortex). Units number refers to the total number of units recorded in selected brain regions after quality control filtering (see Methods). **(D)** The presentation of stimuli in the two datasets (‘Brain Observatory 1.1’, or dataset1, and ‘Functional Connectivity’, or dataset2) is illustrated, where different colours represent different types of stimuli, including gabors, flashes, and natural movies, among others. White blocks represent blank screen. Natural movie 1 (NM1), represented by the light purple blocks, includes two presentation blocks in each dataset. **(E)** 30 images from NM1, each capturing a specific moment within a 1-s bin. The video has a frame rate of 30 fps. **(F)** Neural activity in response to the presentation of image 1 in the example session (ID: 791319847). Right: The corresponding index of units per brain region. Left: The average spiking activity of units in bins of 1 s. **(A,C)** Adapted from Allen Brain Institute and Neuropixels Visual Coding – White Paper v1.0 (October 1, 2019) and Cheat Sheet Version 1.1 (November, 2019).

We analysed a publicly available dataset from the Allen Brain Institute (Siegle et al., 2021). The dataset comprises recordings from a total of 60 mice. In the data recording, the mice were head-fixed but could move on a rotating platform while at the same time showing a set of standardised visual stimuli (Figure 1A). Neuronal activities were recorded simultaneously from up to 6 Neuropixel probes (374 data channels, sampling rate 30 kHz) across various mouse brain areas (Jun et al., 2017), including the primary visual cortex (VISp or V1) and the higher visual areas VISl, VISrl, VISal, VISpm, VISam as well as the thalamus, midbrain regions, and hippocampus (Figures 1B,C). We rendered the activity of units in 1-s bins. During the recording, mice were shown two possible stimulus sets: ‘Brain Observatory 1.1’ (referred to as dataset1) and ‘Functional Connectivity’ (referred to as dataset2) (Figure 1D). In each dataset, natural movie 1 (30 s long, 30 fps) is presented multiple times in each presentation block (10 and 30 repetitions for dataset1 and dataset2, respectively). Snapshots from every second of the movie (30 images in total) are shown in Figure 1E. For an example individual session, the neuronal activity of all units in response to the first frame of the movie across all 20 repetitions is shown in Figure 1F. Different units index correspond to different brain regions, as shown in Figure 1F (left). Our study primarily focuses on the data from dataset1, examining the neural responses to the natural movie stimuli across the various brain regions.

**Figure 2:**
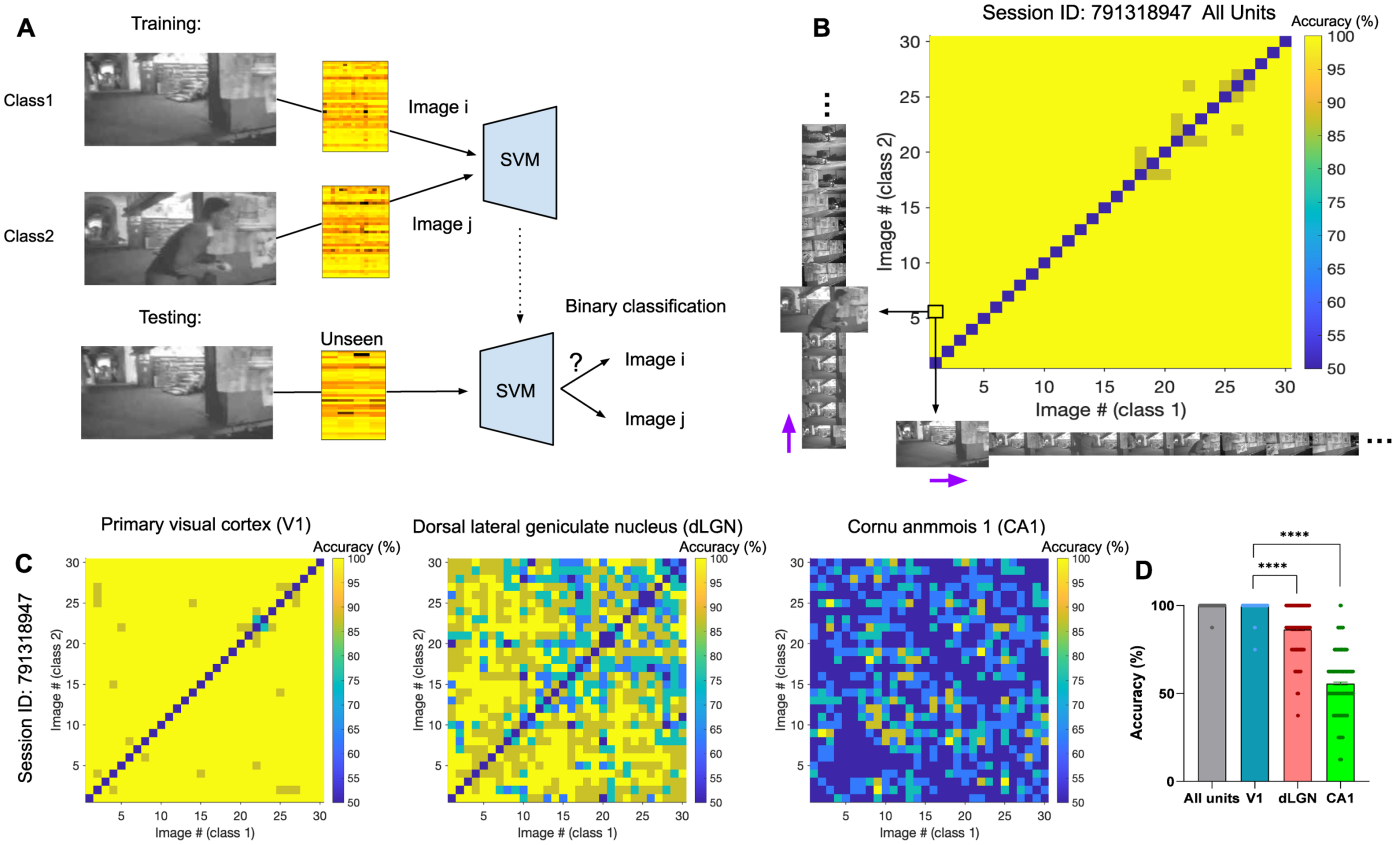
The identity of natural frames can be distinguished from neural activity using linear decoders. **(A)** Illustration of the training (top) and testing (bottom) processes for classification between two example image frames (the first and the sixth frame here). **(B)** The matrix of pairwise classification accuracy when the decoder is trained and tested on neural activity from all units. Accuracy between all pairs of image frames is shown here (x-axis: class1/target image; y-axis: class2/distractor image). The black arrows highlight the example pair of images (image 1 and 6) represented as class 1 and 2 in **(A)**. The purple arrows indicate the sequential direction of natural movie frames. The chance level for the binary classification is 50%. The diagonal represents the accuracy when both class1 and class2 are the same image, which is expected to be at the chance level. **(C)** Specific matrices of pairwise classification accuracies when the decoder is trained and tested on neural activity from specific regions (Left: primary visual cortex (VISp/V1); Middle: dorsal LGN (LGd/dLGN); Right: hippocampal region CA1), in the same session. **(D)** The distribution of the data (dots) from matrices in **(C)**, along with their mean values (indicated by bars). Values on the diagonal are excluded. Error bars show mean ± s.e.m. For statistics, the Kruskal Wallis test was performed to compare multiple groups, **** value of *p* <0.0001.

We first asked how different frames of the natural movie can be decoded from neural activity. To this end, a support vector machine (SVM) decoder was trained to perform the binary classification between pairs of natural movie frames. Specifically, each classification task involved a target image, or class1, and a distractor image, or class2. For each pair of image frames (i-th and j-th), we trained the decoder on 80% of the neural activity to distinguish the two images (i-th and j-th) from each other. The training was performed on neural activity taken from randomly chosen repeats of the movie. We then tested the decoder on the remaining 20% of the neural data and evaluated the accuracy of decoding. The average decoding accuracy was obtained as the mean accuracy across multiple randomizations of the train and test datasets and was compared to the chance level for the binary classification (50%). An example of the classification between the first and the sixth frames (i = 1, j = 6) is illustrated in Figure 2A. The classification accuracy can be visualised for all pairs of image frames by a matrix of pairwise classifications (Figures 2B,C). We presented the results when the decoder uses the activities of all units (Figure 2B) or when the decoding was based solely on the activity of units recorded from specific regions (Figure 2C). We then calculated the average accuracy across all possible image pairs illustrated in the accuracy matrices (Figure 2D), by selecting the upper or bottom triangular half of the matrix in order to avoid counting image pairs twice. The diagonal pairs are excluded. In the three example regions shown, the primary visual cortex (V1) reveals the highest classification accuracy, with an average accuracy approaching 100% (Figure 2D, ‘V1’). The dorsal lateral geniculate nucleus (dLGN) also shows very high levels of accuracy, although lower than V1 (Figure 2D, ‘dLGN’). The neural activity in the CA1 region, in contrast, shows levels of accuracy close to chance levels (50%), suggesting the presence of low or no visual information in the neural activity (Figure 2D, ‘CA1’). Visual areas, in general, show very high levels of classification accuracy (e.g., V1 and dLGN). These results, therefore, suggest that different natural movie frames can overall be distinguished with very good levels of accuracy from neural activity, but different regions contain different levels of visual information.

### Decoding natural movie frames across different brain regions

Next, we applied our classification approach to systematically examine the amount of visual information present in distinct brain regions included across all recording sessions of dataset1 (*n* = 32). In addition to the binary classification, we also trained a multi-class SVM decoder to classify all 30 frames of the natural movie. In this case, we employed a ‘one-*vs*-all’ strategy for multi-class classification. 30 distinct binary classifiers were trained. Each classifier was designed to differentiate a specific frame of the movie (class1) from all the other frames (class2). For each recording session, we randomly selected 80% of the movie repetitions for training, with the remaining 20% used for testing the accuracy of classification. The outcomes were compared with the chance level (50% for binary SVM and 0.11% for multi-class SVM) to assess the performance of classification. A total of 51 brain regions were identified and analysed, spanning the visual cortex (10), hippocampus (9), thalamus (20), midbrain (9), and other regions (3; Supplementary Table 1). The number of units varies in different areas (see Figures 1C,F). We initially tested the impact of units number on decoding performance in four selected brain regions (V1, dLGN, CA1, APN) by randomly selecting 25 units (starting from 1 and increasing incrementally) and averaging the results over 10 recording sessions (Supplementary Figure 1A). Expectedly, as the number of units increased, more visual information was captured by the decoder, with the exception of the CA1 region (Supplementary Figure 1A). However, the impact on decoding accuracy plateaued after reaching a certain threshold (~20 units). We therefore used this threshold to run the decoders and compare the results for different regions. Across all sessions, 30 brain regions were found that met this condition. Decoding was performed on 20 randomly chosen units in each brain region, and the results were reported for the average accuracy across different sessions. Brain regions with fewer than 20 units were excluded from the analysis here (the full results are shown in Supplementary Figures 1B,C).

**Figure 3:**
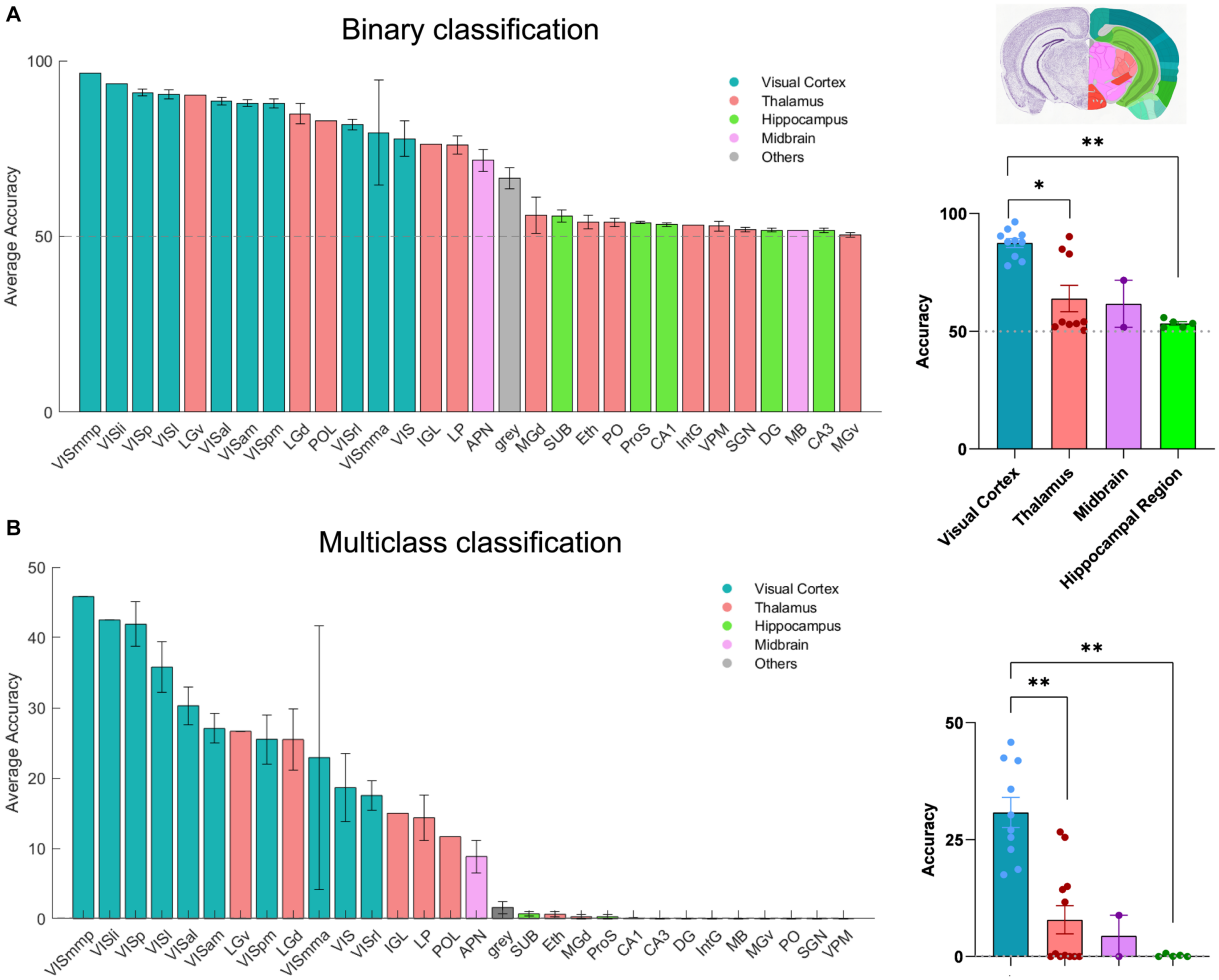
The decoding of neural activity in different brain regions. **(A)** Decoding accuracy of binary classification (20 units), mean ± s.e.m. Left: The bars display the accuracy for each session that includes the given brain region. Right: Scatter plots summarising the overall results for brain regions, which are classified by the source (visual cortex, thalamus, hippocampus, midbrain, and hypothalamus). *n* = 32 recording sessions. For statistics, the Kruskal Wallis test was performed to compare multiple groups, * value of *p* <0.05 ** value of *p* < 0.01. **(B)** Decoding accuracy of multi-class classification (20 units), mean ± s.e.m. For statistics, the Kruskal Wallis test was performed to compare multiple groups, ** value of *p* < 0.01. The grey dashed line shows the chance levels (50% in binary SVM and 0.11% in multi-class SVM; see Methods for details).

The results of the two classification approaches are exhibited in Figures 3A,B. For each classification, the regions are ordered according to the respective average accuracy over all recording sessions, illustrating the variable coding capacity of neural activity across different brain regions. Remarkably, the V1 exhibited exceptional accuracy (~90% in binary and ~ 40% in multi-class), outperforming other regions. The primary mediomedial (VISmmp) and laterointermediate (VISli) areas also demonstrated high accuracy, suggesting they potentially play an important role in decoding. However, it is worth noting that while the performance of V1 was consistent across multiple sessions, VISmmp and VISli were only recorded in only one session.

Other regions of the visual cortex, including the anterolateral (VISal), lateral (VISl), anteromedial (VISam), posteromedial (VISpm), anterior medial (VISmma), and rostrolateral (VISrl) areas, also displayed relatively high accuracy. In the thalamus, the dorsal lateral geniculate nucleus (dLGN) and the ventral lateral geniculate nucleus (vLGN) showed good decoding accuracy and even surpassed some areas of the visual cortex, reflecting their pivotal role in visual information processing.

Additionally, the anterior pretectal nucleus (APN), which is part of the midbrain region, displayed relatively high accuracy. Our results suggest that APN may contain a certain level of visual information. Moreover, most regions within the hippocampus, which is usually considered the vital memory system in the brain, displayed decoding accuracy close to the chance level. However, CA1 demonstrated a slight increase in decoding accuracy as the unit increased (Supplementary Figure 1A) and demonstrated a higher accuracy than CA2 and CA3 (Supplementary Figures 1B,C). Overall, our results demonstrate the differential coding capacities of neural activity across various brain regions.

### Impact of different behavioural states on decoding

We then asked how different behavioural states of the animals may affect our decoding results. To answer this question, we established two distinct groups for comparison. The control group was based on the random selection of movie repeats for training and testing. This was compared with the selection of movie repeats based on the behavioural states of animals, as assayed by the locomotion speed. The behavioural variant group was divided into repeats of high (> = 2 cm/s) and low locomotion speed (<2 cm/s) based on the average running speed. The choice of the speed threshold (2 cm/s) was based on the experimental distribution of running speeds, whereby values below 2 cm/s show a local maximum in the probability density, implying two distinct behavioural states (Supplementary Figure 2; see Methods for details). Binary SVM decoders were trained using 80% of the repeats exhibiting either high (H) or low motion speed (L), with the remaining repeats used for testing.

**Figure 4:**
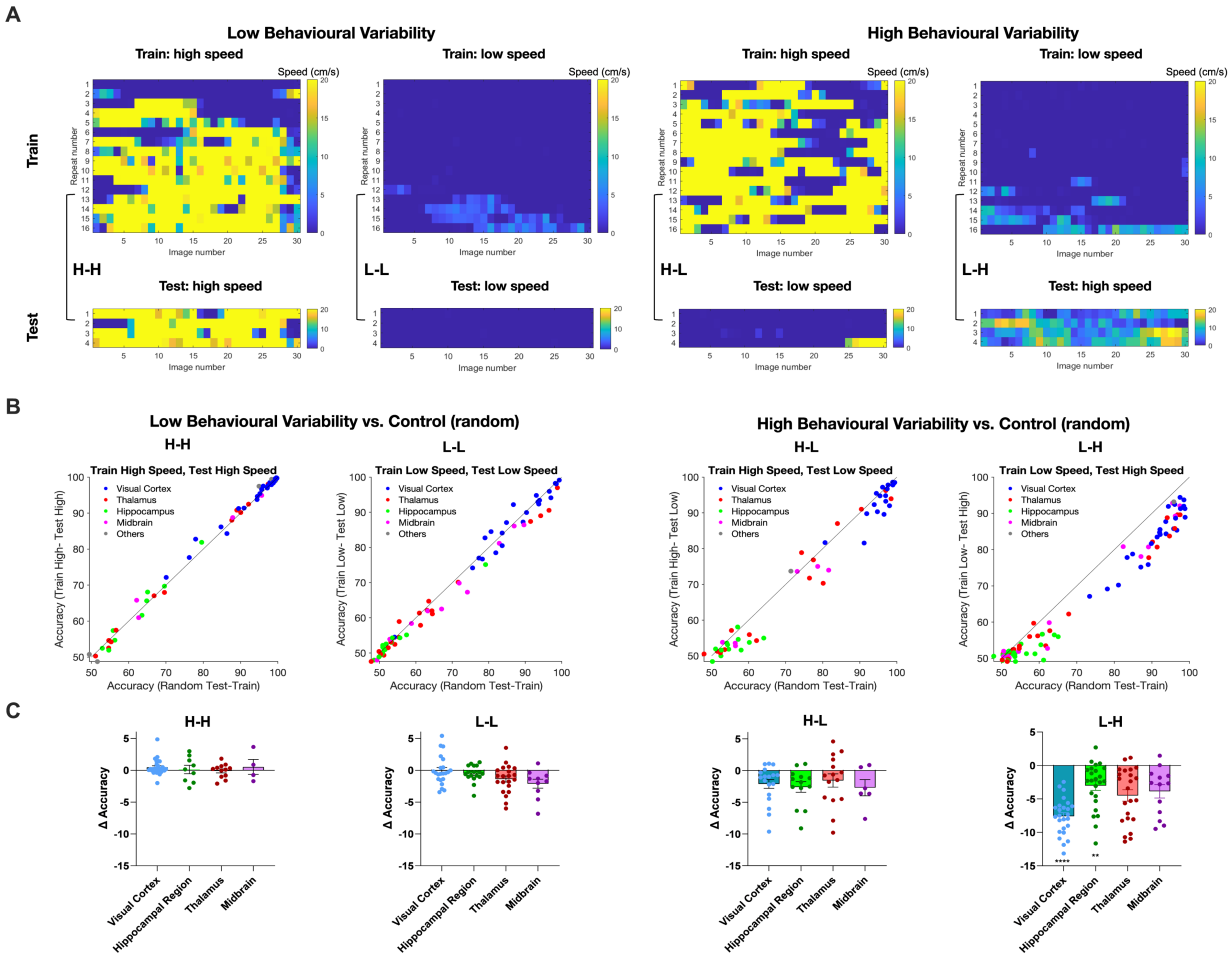
Impact of animal behaviour states on decoding accuracy across different brain regions. **(A)** Examples of recording sessions that were chosen for training (80%) and testing (20%) of the decoder in different combinations. The high-speed (H) or low-speed (L) repeats used in the test set are the highest or lowest average speeds in the session, respectively. The training set uses the remaining repeats. Low behavioural variability conditions correspond to sessions with similar training and test conditions (high-high, H-H; or low-low, L-L, respectively). High behavioural variability conditions correspond to sessions with different training and test conditions (high-low, H-L; or low-high, L-H, respectively). **(B)** The distribution of decoding accuracy across different brain regions for different behavioural conditions vs. control. **(C)** Scatter plots of the difference in accuracy between behavioural conditions and control, for different brain regions. Error bars indicate mean ± s.e.m. For statistics, the Mann Whitney U test was performed to compare two groups (control and behavioural variant group), ** value of *p* < 0.01, **** value of *p* < 0.0001.

Therefore, we generated four possible combinations of behavioural states of training and testing (H-H, L-L, H-L, and L-H; where the former behavioural state is used for training and the latter for testing). We define high behavioural variability as a condition where the training repeats and the test repeats correspond to different behavioural states (H-L and L-H). On the other hand, low behaviour variability refers to the conditions where the training repeats and the test repeats share the same behavioural state (L-L and H-H). To avoid overfitting and to ensure the robustness of our decoding model, we screened all recording sessions and preserved only those that complied with our training and testing scheme (i.e., 80% of data for training and 20% for testing). Examples of such sessions are illustrated in Figure 4A.

For each brain region, we calculated the accuracy of binary classification for each combination of behavioural states and compared it with the control group (Figure 4B). Under low behavioural variability conditions, the decoding accuracy across most regions remained relatively unchanged (Figure 4B). Interestingly, the decoding accuracy of visual regions increased during H-H states, compared to L-L states (Figure 4B). This suggests that visual information increases during running.

In contrast, running had a different effect when train and test repeats were not behaviourally matched. In both H-L and L-H conditions, corresponding to high behavioural variability, most regions displayed decreased decoding accuracy compared to the control (Figure 4B). The effect was, however, more significant when the decoder was trained on L and tested on H states. The most susceptibility to behavioural state alterations was shown in the visual cortex. Decoding outcomes within the visual cortex were significantly decreased by changed behavioural states in L-H (*p* < 0.0001), and the effect was close to significant in H-L (*p* = 0.10) (Figure 4C). Intriguingly, despite the inherently lower decoding accuracy of hippocampal regions, the behavioural state appeared to modulate their decoding accuracy as well, especially in L-H (*p* = 0.0043). None of the changes in decoding accuracy were statistically significant for low behavioural variability vs. control (Figure 4C). We also did the same analysis for V1 units only and observed qualitatively similar results, although the overall accuracy was higher on average (Supplementary Figure 3). These results, therefore, show the effect of behavioural state changes on the decoding of visual information: higher running speed increased the decoding performance, while higher behavioural variability decreased the decoding accuracy.

### Encoding of stimulus and behavioural parameters

Our results demonstrated that natural images can be better decoded from the neural activity when behavioural variability is low between training and test datasets. Which type of encoding model can explain this finding? One possibility is that changes in the behavioural state scale the stimulus-evoked neural responses (e.g., higher running speed or larger pupil size increase the response gain of units). But under such a transformation, a decoding model trained on the L state should be transferable to H state. Another possibility is that the behavioural parameter contributes independently to neural modulation (Sadeh and Clopath, 2022). Under such a transformation, training a decoding model under L state may not translate to H state, as the decoder cannot learn the behavioural modulation in the absence of behavioural inputs in the L state.

To better understand how neural activity is jointly determined by stimulus and behavioural parameters, we therefore turned to the encoding models. We employed Generalised Linear Models (GLM) to predict the neural activity of V1 units from stimulus and behavioural variables. We chose units in the primary visual cortex (V1) as they contain more visual information and higher capacity in decoding images from neural activity (Figures 2, 3). We quantified the contributions of image frames (as stimulus parameters), the animal’s running speed (as animal behavioural parameter), and an interaction term representing the interplay between the two. If the behavioural parameter scaled the stimulus-evoked responses, we would expect large interaction terms in our GLMs.

**Figure 5:**
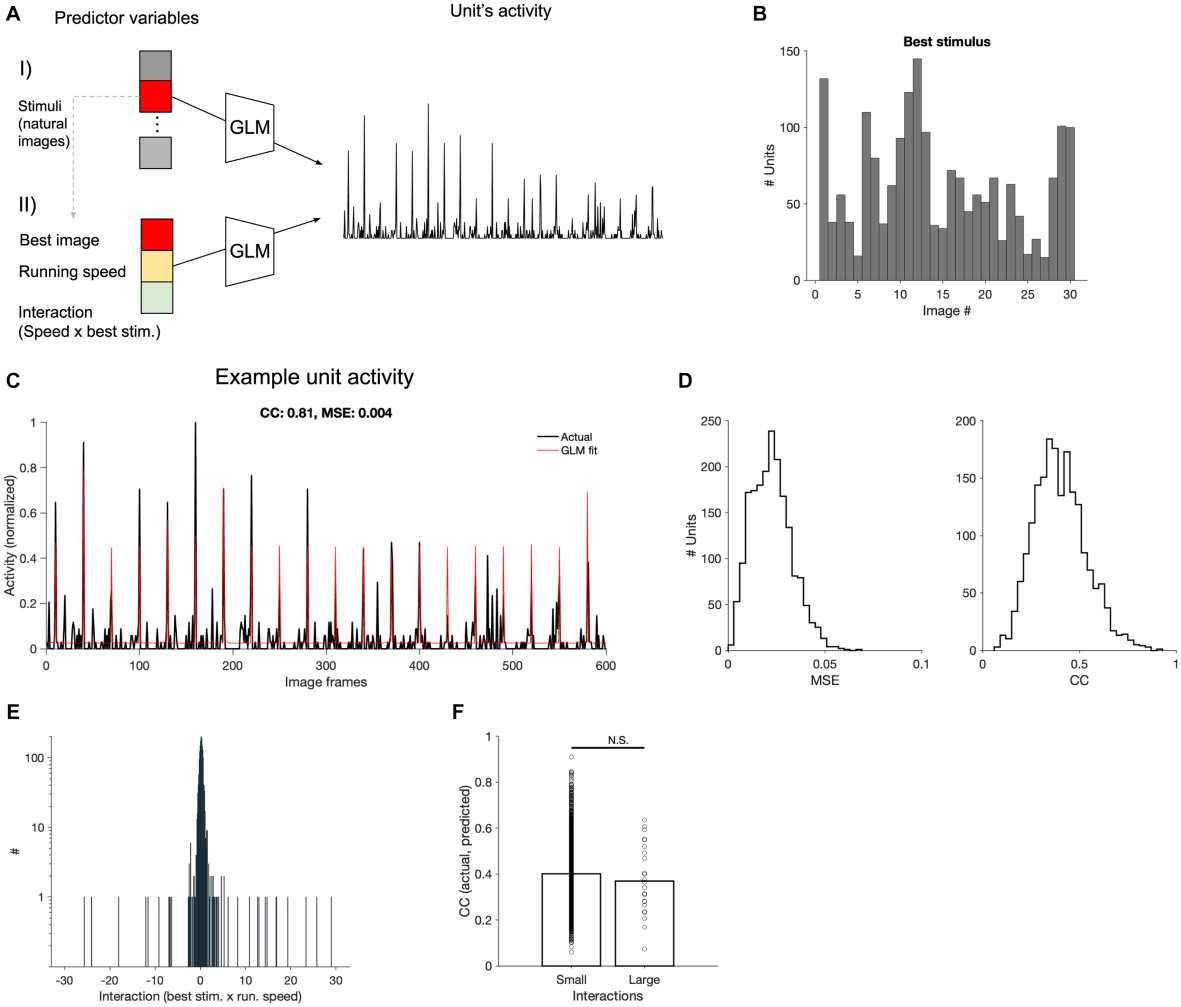
Encoding of stimulus and behavioural parameters. **(A)** Conceptual diagram of the generalised linear model (GLM) employed for encoding. Using the GLM, the stimulus that results in the maximum coefficient for each neuron is determined (I). The maximum stimuli, along with the running speed and its interaction with the stimulus, are used in another GLM to predict neural activity, generating corresponding coefficients (II). **(B)** Distribution of the best stimuli obtained from **(A-I)** across all V1 units. **(C)** Comparison of predicted and actual neural activity obtained from **(A-II)**. The predicted and actual neural activities of a selected V1 neuron are compared across 20 movie repetitions (600 s in total, the correlation coefficient between actual and fitted traces, CC: 0.81; the mean squared error, MSE = 0.004). **(D)** Distribution of the MSE and CC computed from all V1 units across all recorded sessions. **(E)** Distribution of interaction coefficients across all neurons. **(F)** CC between the observed and predicted neuronal firing rates for units with small or large interaction coefficients. The two distributions were not significantly different from each other (value of *p* = 0.25).

To evaluate this interaction between behavioural and stimulus parameters, we pursued a two-stage approach (Figure 5A). In the first step, for each unit, we ran a GLM with only stimulus parameters (30 different natural movie frames), to identify the preferred stimulus (the image with the largest coefficient, indicating the frame that primarily determined the prediction of neural activity; Figure 5B). In the next step, we ran another GLM model with three input parameters: the maximum stimulus (obtained from step 1), the running speed of the animal (as the behavioural parameters), and their interaction term. This process was performed for each V1 neuron across all recording sessions. The GLM models could capture responses of V1 units to various degrees, with some units showing a very good match between actual and fitted responses (Figure 5C). We assessed the goodness of model fits with two metrics: the mean squared error (MSE) and correlation coefficient between actual and fitted traces. There was a distribution of CC and MSE values, with the average of 0.4 for CC and 0.023 for MSE at the population level (Figure 5D).

The coefficients of the stimulus were generally high, suggesting its primary role in driving the predictions of neuronal activity (not shown). Conversely, the coefficients of interaction between running speed and maximum stimulus showed a narrow distribution (Figure 5E), indicating a weak scaling of stimulus-induced activity by running. In fact, there was not a significant difference between the CC of actual and fitted response for units with small or large interaction coefficients (Figure 5F), suggesting that it does not enhance the prediction of responses, on average. These results suggest that the behavioural parameter (i.e., running speed) does not simply scale the stimulus-evoked responses, when animal transitions between different behavioural states (i.e., L and H running speeds). This can explain why our decoding models could not perform as well when trained and tested on neural data belonging to different behavioural states.

### Assessing the visual coding space by ‘decoder receptive fields’

**Figure 6:**
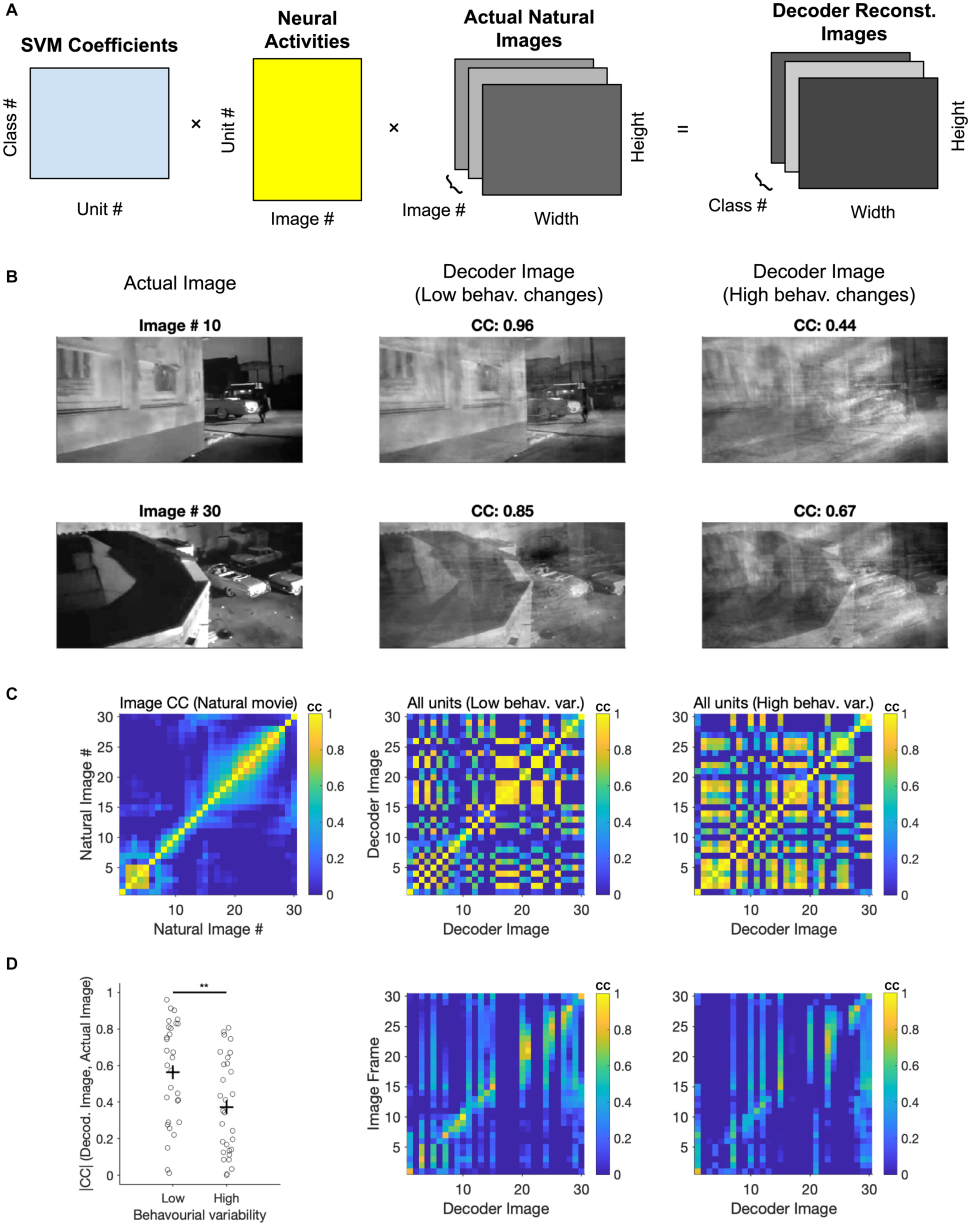
Assessing coding space by reconstructing decoder receptive fields. **(A)** Illustration of the three matrices in the image reconstruction process: The SVM coefficients matrix that assigns weights to each neuron, the activity matrix that represents the neuronal responses of all units to different classes (i.e., different frames of the movie stimuli), and the image information matrix (total image number = 30). These matrices are multiplied together to produce a final 3D array, which is the reconstruction of the natural movie frames. **(B)** Image reconstruction from the decoder. Two examples of image reconstruction from the decoder are shown here. For each example, the decoded image [obtained from the final 3D array produced in **(A)**] is displayed alongside the corresponding actual image from the movie. **(C)** Left, correlation coefficients between actual images. Middle, correlation coefficients between decoder images reconstructed from low behaviour variability sessions (*n* = 3). Right, correlation coefficients between decoder images reconstructed from high behaviour variability sessions (*n* = 3). **(D)** Left, comparison of correlation coefficients between decoder reconstructed images from various behavioural states and the corresponding actual images. Paired t-test was performed, ** value of *p* < 0.01. Middle and Right, Correlation coefficients between the decoder image and the actual image under conditions of low behavioural change (middle) and high behavioural change (right).

We next asked if we could gain further insights into the visual coding space by evaluating the ‘receptive fields’ of the decoders used to distinguish natural images. Since we have employed linear SVMs, the weights (W) with which the multi-class decoder weighs neural activity (A) can be used to reconstruct the image that is ‘seen’ by the decoder. By multiplying these weights by the neural activity of all units to different frames of the movie stimuli (W. A), we can project the neural activity into a low-dimensional space. This space is designed to differentiate between the classes on which the decoder has been trained. These low-dimensional activity projections can then be multiplied with the natural movie (M) to reconstruct the ‘decoder receptive field’ (dRF = W. A. M) for each class (Figure 6A). Note that another way of interpreting this operation is by multiplying the decoder weights (W) with the ‘receptive fields’ of individual neurons (A. M).

We therefore multiplied these three matrices to reconstruct how the decoder represents different frames from a natural movie clip (Figure 6A). The specific matrices included the decoder coefficients for each neuron (across 30 classes), the integrated neuronal activity (averaged from 20 repeats), and specific image information from 30 representative frames of the nature movie. Two examples of reconstructed decoder images are shown and compared with corresponding actual images (Figure 6B). Decoder images showed remarkable similarity to the actual images, despite using a rather simple decoder and only using 50% of repetitions for training (*cf.* 90% train - 10% test in Schneider et al., 2023).

In the above examples, the decoders were trained on the first presentation block (50% of the data). The decoder weights were then used to reconstruct decoder images by using the neural activity from the second presentation block (50%). We performed this for two conditions: when choosing sessions with similar behavioural variability between the first and the second blocks (low behavioural changes), or for sessions with large changes (high behavioural changes). The quality of reconstruction was better for low behavioural changes, as evident from visual inspection and quantified by the correlation coefficient of the reconstructed and actual images in each case (Figure 6B).

We further used the correlation of images to obtain the matrix of similarity both within and across different images (Figures 6C,D). First, we compared the structure of correlation within natural images themselves with the pairwise correlation of reconstructed images from decoding (Figure 6C). The overall structure of correlations seems to be preserved in reconstructed images. However, reconstructed decoding images showed more spurious pairwise correlations under high behavioural changes (Figure 6C). This suggests that they would be less distinguishable in the decoder space and can therefore explain the lower accuracy of classification under high behavioural variability.

We also quantified the similarity of reconstructed images with actual images by calculating the matrix of correlations between the respective images. There was a range of reconstruction qualities, but many images could be reconstructed with high accuracy (Figure 6D). Importantly, reconstructing images under high behavioural variability led to decreased correlation with the real images compared to low behavioural variability (*p* = 0.0086), consistent with our results before (*cf.* Figure 4). Taken together, our results shed further light on how behavioural variability may affect the results of decoding natural images. More generally, the results of this analysis suggest that by reconstructing the low-dimensional decoding images, we can obtain key insights into the visual coding space when recording high-dimensional neural responses.

## Discussion

### Natural visual stimuli can be decoded from neural activity

Our results suggest that visual information can be decoded from neural activity, but it varies depending on different brain regions and behavioural states of the animal. In particular, using SVM decoders with linear kernels, we classified different frames of a natural movie from neural activity. This allowed us to interpret the decoding results by projecting the high-dimensional neural activity over the low-dimensional space of classification, which is most likely relevant to animals’ perception, decision making and behaviour.

### Decoding of natural images from neural activity depends on brain regions

Differences in decoding accuracy between brain regions underlie regional specificity in visual information processing. We demonstrate that the visual cortex, especially the primary visual cortex (V1), plays a central role in decoding visual information. Moreover, lateral geniculate nuclei (dLGN and vLGN) showed the best decoding accuracy in the thalamus region, which is consistent with the fact that they are upstream of V1 and receive visual information from the retina (Tong, 2003; Jeffries et al., 2014; Covington and Al Khalili, 2023). The midbrain regions, APN, also show relatively high accuracy. Even though the role of APN in sensory processing has been established, it has not been associated with direct retinal inputs or connections to the visual accessory nuclei (Rees and Roberts, 1993). One possibility could be that this is because the midbrain could interact with the thalamus to manage attention (Knudsen, 2011), especially the visual selective attention, and shape high-level visual properties (Bogadhi et al., 2021) (however, see our discussion below in ‘Limitations of our study’).

### Behavioural variability affects the results of neural decoding

By decoding the visual content from the neural activity of mice in different behavioural states, our results revealed that behavioural variability can significantly affect the accuracy of decoding. When train and test datasets were chosen with high behavioural variability (high running speed for train and low running speed for test, H-L; or low running speed for train and high running speed for test, L-H), the classification accuracy was reduced compared to control data, where train and test datasets were chosen randomly. Within those two conditions, L-H led to stronger decreases in decoding accuracy, suggesting that the decoder cannot learn about the modulation of neural activity by behaviour in the H test state, when it is trained on L train data. Under low behavioural variability, namely, when we used similar behavioural states (L-L or H-H), the decoding accuracy was similar to the control. However, the decoding accuracy was higher in H-H, suggesting that running could increase the decoding accuracy, if the decoder is trained on datasets with running.

### Encoding of stimulus and behavioural parameters

The results of our encoding modelling shed further light on the interaction of stimulus and behavioural parameters in modulating neuronal responses. We chose the generalised linear models (GLM), which is a common and effective encoding method in understanding and modelling neural responses (Desbordes et al., 2010; Shi et al., 2015; Hosseini et al., 2021; Williams et al., 2023), to predict the neural activity of units in V1. Our results showed that visual stimuli contribute significantly to predicting neural activity. Moreover, our results revealed weaker interaction of behavioural and stimulus parameters in predicting neural activity. Our analysis therefore suggests that behavioural states do not simply scale natural movie-evoked neural responses but may influence neural activity through more complex mechanisms (Erisken et al., 2014; Dadarlat and Stryker, 2017). These mechanisms may involve different subtypes of interneurons as well as the effects of locomotion (Garcia del Molino et al., 2017; Dipoppa et al., 2018).

Our study employed a binary vector to represent our stimuli, where only the frame currently being stimulated was represented as 1 and all others as 0. Each frame was uniquely encoded to ensure its stimuli were solely responsible for influencing the model’s predictions. This approach may only partially consider the complex correlation among visual stimuli (natural movies). Recent research suggests that a nonlinear mixed selectivity coding scheme, where multiple stimulus features are conjunctively coded, may provide a more comprehensive and reliable encoding of visual stimuli (Johnston et al., 2020). Besides, a model containing semantic information may increase the model’s explanatory power for brain activity (Naselaris et al., 2009; Takagi and Nishimoto, 2022). It would be interesting to see if including these features in more advanced decoding models can improve the accuracy and quality of decoding.

### Image reconstruction from decoders and the low-dimensional visual coding space

Since we used SVM decoders with linear kernels, our approach could effectively reconstruct the images ‘seen’ by the decoders to distinguish between different images. This is subject to the classification problem the decoder is trying to solve, as this shapes the low-dimensional space within which the decoder needs to maximise the distance of high-dimensional population responses. Therefore, our multi-class SVM, which was trained across 30 classes (individual movie frames), could obtain a more realistic weight distribution for each participating neural unit. We obtained a high average correlation between our reconstructed images and the actual images (average CC = 0.6; Figure 6), explaining why the multi-class SVM was capable of distinguishing the majority of the images with good accuracy (Figure 2).

Consistent with our results on the dependence of the accuracy of decoders on behavioural variability (Figure 4), we also found that high behavioural variability resulted in lower quality of reconstructed images. It would be interesting to see how the animals manage to correct for significant behavioural changes to reach the same levels of decoding accuracy. This might be achieved by training the decoders on high behavioural variability (as we showed similar levels of accuracy for H-H states), by focusing on reliable units (Sadeh and Clopath, 2022), and/or by employing predictive coding (Friston, 2010; Keller and Mrsic-Flogel, 2018; Hogendoorn and Burkitt, 2019). It would also be interesting to apply recently developed encoding-decoding methods (Schneider et al., 2023) to see how behavioural and neural activity data can be mutually combined to inform a robust decoding of natural images, independent of behavioural states. It would be important to try these approaches on individual sessions/animals, as concatenating the data into ‘super mice’ (Deitch et al., 2021; Schneider et al., 2023) averages over individual variability. Future work should test these advanced methods on sessions with high behavioural changes (*cf.* Figures 4, 6) to see if the decoding accuracy can be improved in individual animals.

Instead of retrieving the identities of natural movie frames (see Schneider et al., 2023), here we reconstructed the actual images from our decoders. This was achieved due to the employment of linear decoders, which are more interpretable, as opposed to nonlinear methods (Schneider et al., 2023). However, we reconstructed the decoder images by using the limited number of natural movie frames shown to the animal. Approximating the decoder receptive fields with limited images is comparable to obtaining the receptive fields of units with a limited number of stimuli, rather than mapping them with detailed and fine-tuned stimuli (*cf.* Figure 6A). In future work, it would be interesting to map the neuronal receptive fields by presenting them with noise or Gabor patterns (Jones and Palmer, 1987; Kay et al., 2008), which has been performed within the same datasets (Siegle et al., 2021) as visual stimuli. This may provide a more refined reconstruction of decoder images, and how individual neurons, with their unique receptive field properties, contribute to the overall decoding of visual stimuli.

### Limitations of our study

In our analysis, we relied on the spike sorting and registrations performed by the Allen Brain Observatory, based on their established procedures. However, it is possible that this procedure is not perfect, and there might be inherent limitations or errors in the spike sorting and registration process. These potential challenges could influence our results and the interpretation of our findings. For instance, higher than expected visual information we found in APN might be due to misclassification of the units. It would be interesting to repeat our analysis on the same data with more recent methods (Fabre et al., 2023), and see if the results hold. Additionally, there are inherent limitations posed to our analysis based on the number of units. Some brain regions, such as VISmmp and VISli, have a limited number of recordings across all sessions in the analysed datasets. Therefore, the results from these regions should be interpreted with caution. Future studies with more units across multiple regions should enable a more consistent comparison of decoding across brain regions. To achieve more robust and consistent results, increasing the number of recordings and ensuring a minimum number of recorded units (~20–50) for each region is suggested. More number of stimulus presentations may also be useful in future analyses, as longer blocks of stimulus presentation (with more repetitions) may increase the behavioural variability and hence aid the analysis.

## Conclusion

In summary, we provided a systematic survey of how natural movie frames can be decoded from neural activity in multiple brain regions and across behavioural states. By quantifying decoding accuracy and reconstructing decoders’ receptive fields, our study suggested a computational approach to analyse and visualise the low-dimensional structure of visual coding within high-dimensional neural activity patterns. Our decoding approach, combined with encoding analysis, can help to obtain a comparative picture of the spectrum of visual information present in different areas, and how this can be modulated by behavioural states.

## Methods

### Data acquisition and pre-processing

Dataset from the Allen Brain Observatory (Siegle et al., 2021) was accessible through the AllenSDK.^1^ The data in our analysis constituted 50 sessions distributed across two distinct datasets based on the presentation sequences: the brain observatory dataset (referred to as dataset 1, *n* = 32 sessions) and the functional connectivity dataset (referred to as dataset 2, *n* = 18 sessions). During the recording, mice were exposed to a range of stimuli (including natural movies), each with varying durations and presentation sequences across the two datasets.^2^

Each recording in the dataset measured multiple regions (Figure 1), conducted simultaneously with up to 6 Neuropixels probes (374 data channels, sampling rate 30 kHz; Jun et al., 2017). The spike sorting and registration procedures were carried out by the Allen Brain Institute. Kilosort2 was used (Stringer et al., 2019) and included units that passed the default quality standards. We excluded any invalid intervals that were denoted as not a number (NaN) value. For more detailed information about the experiment preparation, visual stimuli, data acquisition, and data preprocessing steps, please refer to the Technical White Paper from the Allen Brain Observatory on Neuropixels Visual Coding.

In this study, we chose a specific stimulus, namely ‘natural movie 1’. The activity was recorded from the onset of the presentation of each stimulus block. These presentations were divided into two blocks (each block, 10 repeats in dataset1, 30 repeats in dataset2). The first half of the repetitions of this movie stimulus in each dataset was classified as block 1, and the second half of the repetitions were classified as block 2. For analysis, we partitioned the spiking activity of the neurons into bins of 1 s each. Behavioural information (including speed) was obtained in the same time intervals.

### Data normalisation

For the GLM analysis, we applied normalisation procedures to both neural activity and motion speed data. For the neural activity, we normalised by min-max:


$$\begin{array}{l} \text{Normalised}\;\text{activity}=\\ \left(\text{activity}-\min\;\text{activity}\right)/\left(\max\;\text{activity}-\min\;\text{activity}\right)\;, \end{array}$$

where activity is the original neuronal spike count. We also normalised the locomotion speed by min-max:


$$\begin{array}{l} \text{Normalised}\;\text{speed}=\\ \left(\text{speed}-\min\;\text{speed}\right)/\left(\max\;\text{speed}-\min\;\text{speed}\right)\;. \end{array}$$

### Different behavioural states

We selected the locomotion speed of the animal as our behavioural variable of interest to investigate the impact of behavioural variability on neural decoding. The average locomotion speed of the anima during each repeat of the natural movie was used to categorise the state of the movie presentation as either low (L) or high (H) speed. Movie presentations with an average speed of <2 cm/s were categorised as low-speed repeats, and repetitions of the movie with an average speed of > = 2 cm/s were categorised as high-speed repeats.

### Support vector machine decoders

We used Support Vector Machines (SVM) as our supervised decoding models to understand how neural responses map to external stimuli. Our decoder was implemented with a linear kernel SVM, which is particularly suitable for decoding tasks involving high-dimensional input features, such as neuronal firing rates. The implementation was done in The MathWorks Inc. (2021).

The performance of the decoder was evaluated using cross-validation. In this approach, the data was divided into training and test sets. For the experiments depicted in Figures 1–3, we used 80% of all repeats as the training set, which were selected either randomly or based on behavioural states. The remaining 20% served as the test set. In the case of Figure 5, the data was split evenly, with 50% serving as the training set (block1) and the remaining 50% as the test set (block2). These training and test sets were completely independent of each other.

The SVM decoder was trained on a binary classification task to distinguish between pairs of natural movie frames (i-th and j-th frames). In the binary decoder, one target class was labelled as the positive category, or class 1, and the other as the negative category, or class 2. After training, the decoder returned a vector of predicted class labels, either positive or negative, based on the trained SVM model.

### Decoder accuracy calculation

We used the classification accuracy, by comparing the results of the SVM predictions with the actual data, to characterise the ability of the brain region to decode the neural activity. For each pair of image frames, we calculate how many of the SVM predictions are correct (100% if all are correct). The positive and negative examples represent the two types of images (one frame and another random frame) in our SVM test, respectively. The accuracy of prediction is therefore given as:


$$\text{Accuracy}=\left(TP+TN\right)/\left(TP+NP+TN+FN\right)$$

where, TP is True Positive (when the model correctly predicts positive samples), FP is False Positive (when the model incorrectly predicts negative samples as positive), TN is True Negative (when the model correctly predicts negative samples), and FN is False Negative (when the model incorrectly predicts a positive sample as a negative sample). For a binary classification task between a positive class and a negative class, the chance level is 50%. The classification accuracy of a session is then computed by calculating the average SVM prediction accuracy after combining all frames in each recording session. The classification accuracy of each brain region is obtained by selecting the recording units corresponding to that brain region and averaging all sessions containing that brain region.

### Multi-class decoders

We trained 30 individual binary classifiers to distinguish between a particular category (class 1/the positive category) and all other categories (class 2/the negative category). After all the SVM models are trained, the decoder uses these models to make predictions on the test set. Specifically, the decoder used each SVM model to calculate its score of belonging to the positive category, and then the category with the highest score was used as the predicted category. For each binary classifier, the ratio between positive and negative categories is 1:29. For each positive category, the classifier has a 1/30 probability of correctly predicting the positive category and a 29/30 probability of incorrectly predicting the negative category. Each binary classifier has a 1/30 probability of correctly identifying the positive category. During prediction, our decoder opts for the class associated with the classifier that demonstrates the highest confidence among the 30 classifiers. Consequently, the chance level of decoding accuracy in this task can be calculated as 1/30 × 1/30 = 1/900 (~0.11%). The performance of the multi-class decoder was evaluated in the same way as described before.

### Generalised linear models (GLM)

The GLM was fitted with a normal distribution. The link function was chosen to be the logarithm, ensuring that the predicted firing rates were always positive. The stimuli applied in this model were represented using a binary encoding scheme: The frame currently being presented is assigned a value of 1, while all other frames are designated a value of 0. For the first GLM fit, the predictor variables included the presented stimuli. The best stimulus (the stimulus with the highest coefficient) was then used to fit another GLM. The predictor variables in this GLM included the best stimulus, the behavioural state of the animal, and the interaction between the two. Each predictor variable was associated with a coefficient, which the GLM estimated to minimise the difference between the observed and predicted firing rates. The following equation can be used to represent the model:


Y=β0+β1X1+βX2+…+βnXn+ε,

where Y is the predicted variable, such as the firing rates of neurons. X1,X2,…,Xn , are predictor variables, which could include the presented stimuli, the behavioural state of the animal, and other variables. β0,β1,…,βn are the associated coefficients, estimated by the GLM to minimise the difference between the observed and predicted firing rates. ε is the error term, representing the difference between the predicted and actual values.

### Evaluation of predicted firing rate by mean squared error

The performance of the GLM encoder is evaluated using the mean squared error (MSE), which is calculated as the mean value of the square of the prediction error:


$$MSE=\frac{1}{n}\Sigma^{n}{}_{i=1}{\left(Y_{i}-\hat{Y_{i}\;}\right)}^{2}$$

where $Y_{i}$ is the true value, $\hat{Y_{i}}$ is the predicted value, and n is the total number of samples.

### Reconstructing the ‘decoder receptive field’ for each class

Our multi-class linear SVM decoder can make predictions by weighting ( W ) the neural activity ( A ) with their corresponding coefficients. We multiply these weights with the neural activity ( W.A ) to obtain a projection of the neural activity. These low-dimensional activity projections can then be multiplied with the natural movie ( M ) to reconstruct the ‘decoder receptive field’ for each class (dRF = W.A.M ). For each reconstructed image, we computed the Pearson correlation coefficient between the image and the corresponding real image as a measure of similarity. This coefficient ranges from −1 (perfect negative correlation) to +1 (perfect positive correlation), with a value of 0 indicating no similarity. A higher correlation coefficient indicates that our reconstructed image is closer to the real image. As the linear decoder is agnostic about the sign of the reconstruction to discriminate between classes, we have taken the absolute value of the coefficients to quantify the similarity.

## Data availability statement

Publicly available datasets were analysed in this study. This data can be found at: https://portal.brain-map.org/explore/circuits/visual-coding-neuropixels.

## Author contributions

YX: Data curation, Formal analysis, Investigation, Methodology, Project administration, Resources, Software, Validation, Visualization, Writing – original draft, Writing – review & editing. SS: Conceptualization, Data curation, Investigation, Software, Visualisation, Writing – original draft, Writing – review & editing, Formal analysis, Funding acquisition, Methodology, Project administration, Resources, Supervision, Validation.

## Footnotes

^1^ https://allensdk.readthedocs.io

^2^ https://allensdk.readthedocs.io/en/latest/_static/neuropixels_stimulus_sets.png

## Supplementary material

SUPPLEMENTARY TABLE S1 List of brain region abbreviations and their full names.

SUPPLEMENTARY FIGURE S1 Decoding analysis when all the recorded units in all brain regions are considered. **(A)** Relationship between the number of units and the decoding accuracy. The decoding accuracy was obtained from binary classifiers after selecting up to 25 units from the thalamus, midbrain, visual cortex, and hippocampus in at least 10 recording sessions and averaged. **(B)** Decoding accuracy of binary classification from all units. Results are presented as mean ± s.e.m. Note that the number of units are different for every region and session. Left: The bars display the accuracy for each session that includes the given brain region. Right: scatter plots summarising the overall results for brain regions, which are classified by the source (visual cortex, thalamus, hippocampus, midbrain and hypothalamus). n = 32 recording sessions. **(C)** Decoding accuracy of multi-class classification from all units. For statistics, the Kruskal-wallis test was performed to compare multiple groups, ***p* value <0.01 ****p* value <0.001. The grey dashed lines show the chance levels.

SUPPLEMENTARY FIGURE S2 Distribution of running speed for two different datasets. **(A)** Left: the distribution of speed in the dataset1, which contains 20 repetitions. Right: the distribution of speed in the dataset2, which contains 60 repetitions. The vertical line (at speed = 2cm/s) denotes the speed threshold chosen to distinguish low and high speed states. Note the logarithmic scales on both axes. Bin length = 1. **(B)** The distribution of speed for each movie frame, combining data from both dataset1 and dataset2. Note the logarithmic scales on both axes. Overall, the distributions look very similar across movie frames; specifically, the bimodal nature of the distributions with a distinct peak at < 2cm/s is preserved.

SUPPLEMENTARY FIGURE S3 Impact of mice behavioural states on decoding accuracy when only units in the primary visual cortex (V1) are considered. **(A)** The distribution of decoding accuracy for the V1 region across different behavioural states versus control. **(B)** Scatter plots showing the difference in decoding accuracy between behavioural states and control for the V1 region. Error bars indicate mean ± s.e.m.
