# Supplementary figures and images for: Computational assessment of visual coding across mouse brain areas and behavioural states
Source: Front Comput Neurosci. 2023 Oct 13;17:1269019. doi: 10.3389/fncom.2023.1269019 (PMC10613063; doi:10.3389/fncom.2023.1269019)

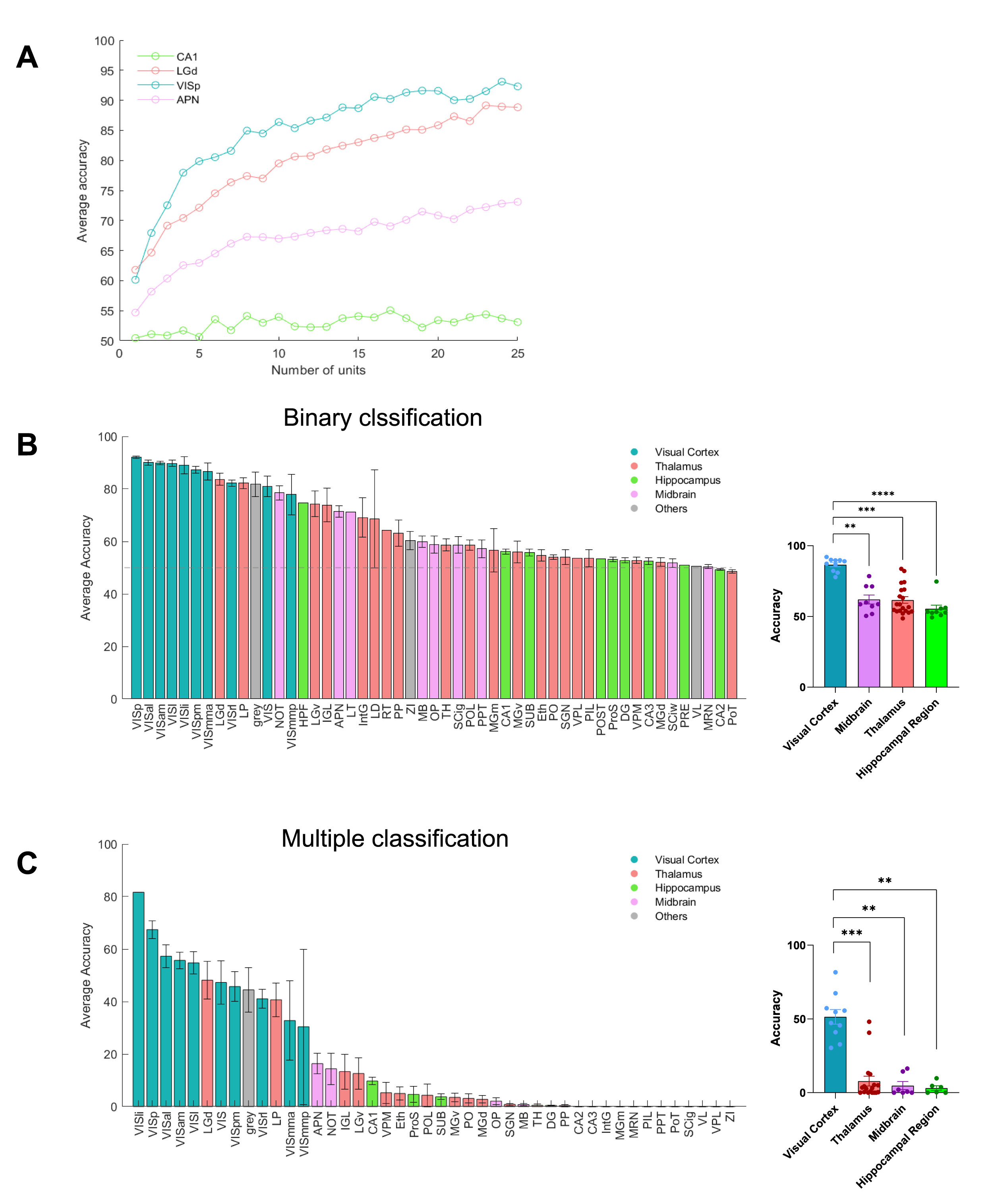

Supplement: SUPPLEMENTARY TABLE S1 — List of brain region abbreviations and their full names. [file Image_1.TIF]

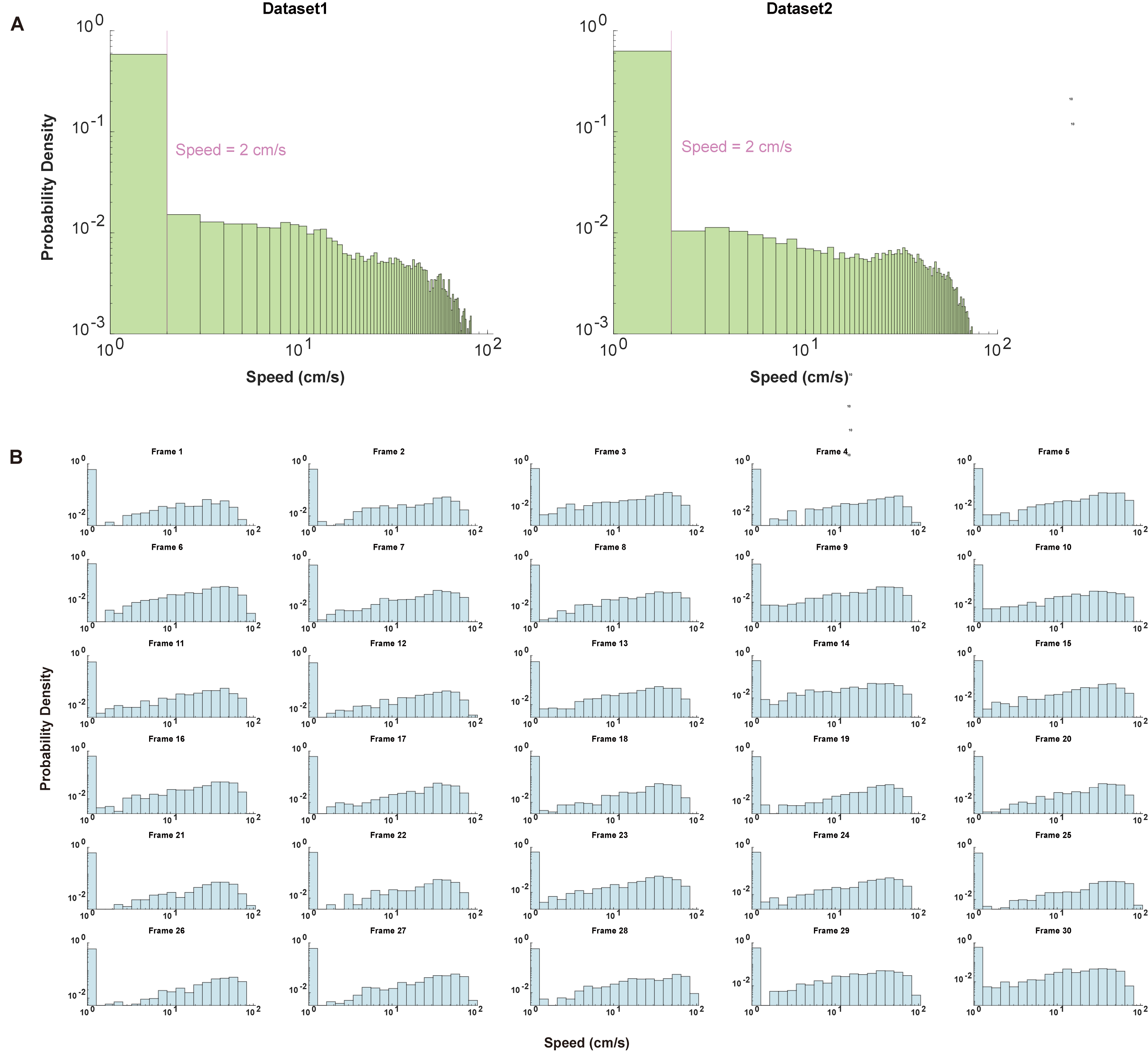

Supplement: SUPPLEMENTARY FIGURE S1 — Decoding analysis when all the recorded units in all brain regions are considered. (A) Relationship between the number of units and the decoding accuracy. The decoding accuracy was obtained from binary classifiers after selecting up to 25 units from the thalamus, midbrain, visual cortex, and hippocampus in at least 10 recording sessions and averaged. (B) Decoding accuracy of binary classification from all units. Results are presented as mean ± s.e.m. Note that the number of units are different for every region and session. Left: The bars display the accuracy for each session that includes the given brain region. Right: scatter plots summarising the overall results for brain regions, which are classified by the source (visual cortex, thalamus, hippocampus, midbrain and hypothalamus). n = 32 recording sessions. (C) Decoding accuracy of multi-class classification from all units. For statistics, the Kruskal-wallis test was performed to compare multiple groups, **p value <0.01 ***p value <0.001. The grey dashed lines show the chance levels. [file Image_2.TIFF]

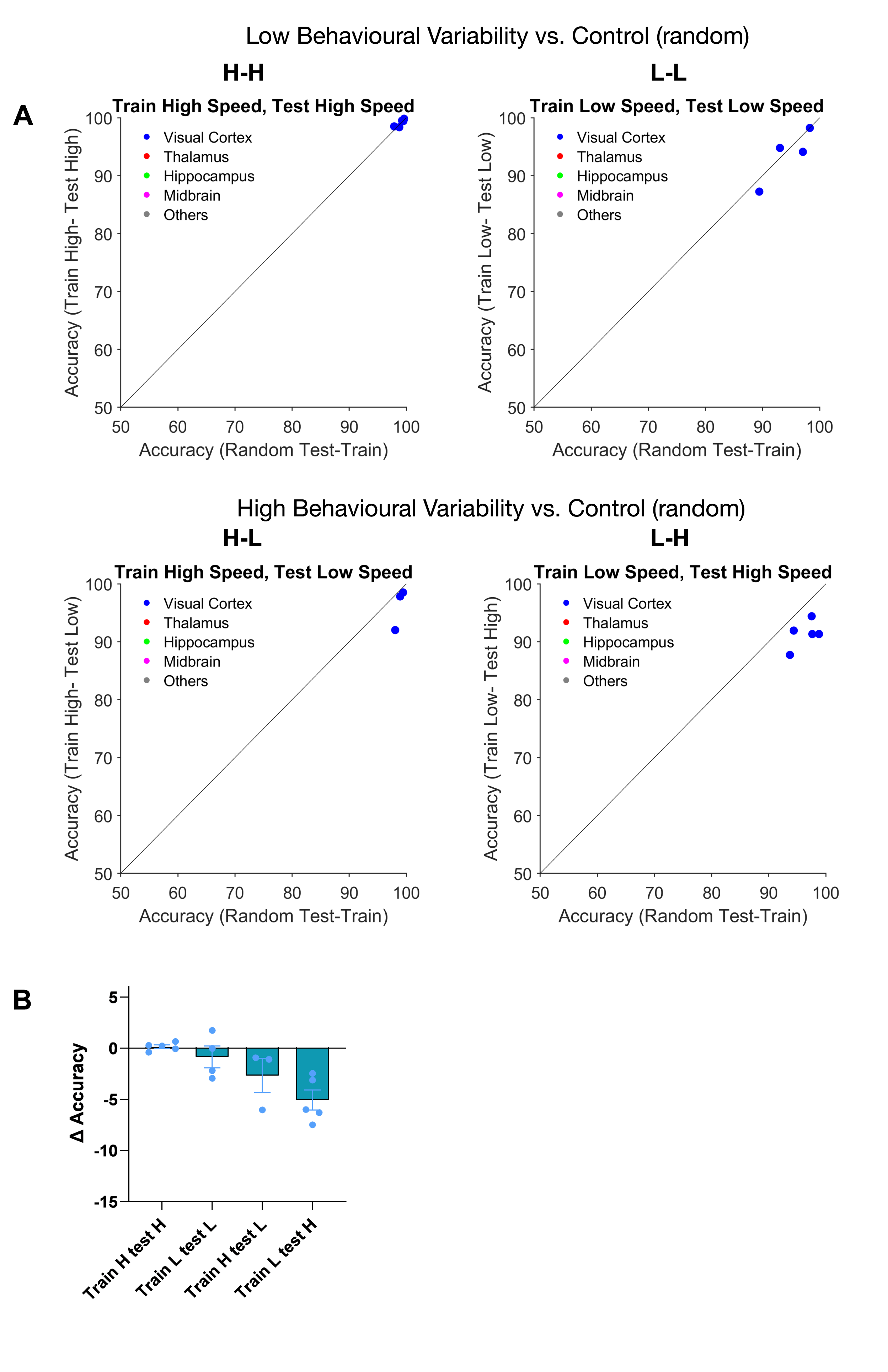

Supplement: SUPPLEMENTARY FIGURE S2 — Distribution of running speed for two different datasets. (A) Left: the distribution of speed in the dataset1, which contains 20 repetitions. Right: the distribution of speed in the dataset2, which contains 60 repetitions. The vertical line (at speed = 2cm/s) denotes the speed threshold chosen to distinguish low and high speed states. Note the logarithmic scales on both axes. Bin length = 1. (B) The distribution of speed for each movie frame, combining data from both dataset1 and dataset2. Note the logarithmic scales on both axes. Overall, the distributions look very similar across movie frames; specifically, the bimodal nature of the distributions with a distinct peak at < 2cm/s is preserved. [file Image_3.TIFF]

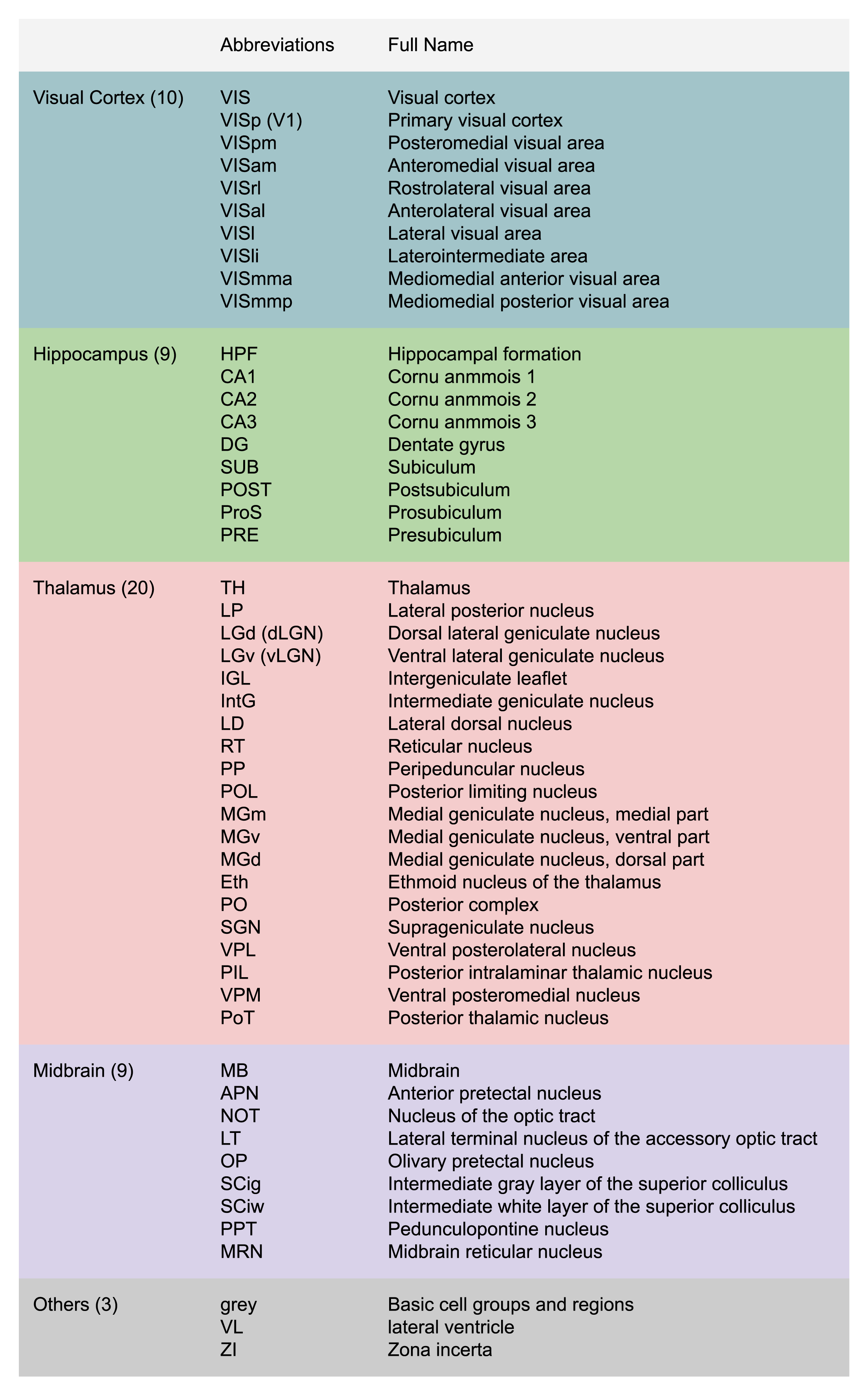

Supplement: SUPPLEMENTARY FIGURE S3 — Impact of mice behavioural states on decoding accuracy when only units in the primary visual cortex (V1) are considered. (A) The distribution of decoding accuracy for the V1 region across different behavioural states versus control. (B) Scatter plots showing the difference in decoding accuracy between behavioural states and control for the V1 region. Error bars indicate mean ± s.e.m. [file Image_4.TIF]
